# Ablation index value for transmural lesions based on unipolar electrograms in patients with paroxysmal atrial fibrillation undergoing pulmonary vein isolation

**DOI:** 10.3389/fcvm.2024.1449623

**Published:** 2024-11-26

**Authors:** Yijun Sun, Binhao Wang, Mingjun Feng, Yibo Yu, Fang Gao, Weidong Zhuo, Yingbo Qi, Xinhui Qiu, Huimin Chu, Guohua Fu

**Affiliations:** ^1^ Arrhythmia Center, The First Affiliated Hospital of Ningbo University, Ningbo, China; ^2^ Health Science Center, Ningbo University, Ningbo, China; ^3^ Key Laboratory of Precision Medicine for Atherosclerotic Diseases of Zhejiang Province, The First Affiliated Hospital of Ningbo University of China, Ningbo, China

**Keywords:** unipolar electrogram, ablation index, paroxysmal atrial fibrillation, pulmonary vein isolation, catheter ablation

## Abstract

**Background:**

It remains unclear whether the current recommended ablation index (AI) value is suitable for individualized catheter ablation. Prior research has established that the elimination of the negative component of the unipolar electrogram (UP-EGM) applications reflects the formation of transmural lesion during radiofrequency ablation. The aim of this study was to explore the relationship between AI values when UP-EGM turns positive during pulmonary vein isolation and recommended AI values.

**Methods:**

A total of 50 patients with drug-refractory PAF who underwent index RFCA were consecutively included from September 2022 to January 2023. All the patients underwent AI-guided ablation. UP-EGM was also recorded during the procedure. The difference in the AI between the value when the UP-EGM turned completely positive [AI_UP-EGM(+)_] and the recommended value at the end of ablation (AI_END_) was compared.

**Results:**

A total of 2 954 lesion points were detected in 50 patients. The average values of AI_UP-EGM(+)_ at the anterior wall and the posterior wall were 420.9 and 267.4, respectively. The average AI_END_ values were 524.3 and 393.9 at the anterior wall and the posterior wall, respectively. The percentage of increase in the AI between the AI_UP-EGM(+)_ and AI_END_ groups was 22%, 28% at the anterior wall and 47%, 49% at the posterior wall (*P* < 0.001). After a mean follow-up duration of 11.30 ± 2.10 months, 44 patients (88%) remained in sinus rhythm without antiarrhythmic drugs.

**Conclusion:**

The AI_UP-EGM(+)_ was lower than the recommended value for all the pulmonary vein regions. The recommended AI value seems to be too high for the posterior and inferior walls, but this remains to be proven in future research.

## 1 Introduction

Pulmonary vein isolation (PVI) is the cornerstone of catheter ablation procedures in patients with paroxysmal atrial fibrillation (PAF) (1). However, long-lasting, continuous, and transmural PVI is still a clinical challenge (2). Therefore, the ablation index (AI), which is a formula incorporating power, contact force and catheter stability, was developed and is widely used to guide radiofrequency catheter ablation (RFCA). Phlips et al. demonstrated the efficiency, safety, and efficacy of the CLOSE protocol using AI-guided PVI (3). However, the thickness of atrial tissue differs across individuals and across regions. Regular AI values are not suitable for all regions. Previous investigations have demonstrated that the elimination of the negative component of the unipolar electrogram (UP-EGM) during radiofrequency (RF) applications reflects transmural lesions (TLs) and is an effective guide for PVI (4–6). Thus, UP-EGM-guided ablation may be more suitable for PAF patients because of its safety, effectiveness and individuation (7). Bortone et al. (8) reported that the termination of ablation at the time of complete positive morphology partly resulted in reversible transmurality, and in such cases, a 5-second extension of ablation was necessary. The aims of the present study were as follows: (1) to explore the difference between the AI when UP-EGM turns completely positive [AI_UP-EGM(+)_] and the current recommended AI value; and (2) to determine the suitable modified AI value based on UP-EGM to guide RFCA.

## 2 Materials and methods

### 2.1 Study population

A total of 50 patients with drug-refractory PAF undergoing index RFCA at the Arrhythmia Center of the First Affiliated Hospital of Ningbo University were respectively included from September 2022 to January 2023. The criteria for exclusion from this study were specified as follows: (1) age <18 or >80 years, (2) the presence of a mechanical mitral valve prosthesis, (3) left atrium (LA) diameter >45 mm, (4) dysfunction of the thyroid gland, (5) left ventricular ejection fraction <40%, (6) intolerance to anticoagulant therapy, (7) current malignancy, (8) life expectancy <1 year, and (9) prior catheter or surgical AF ablation.

The study protocol obtained clearance from the regional ethics review boards and complied with the Helsinki Declaration. Each participant received comprehensive information regarding the research methodology and subsequently granted their informed written consent.

### 2.2 Mapping and RF catheter ablation

The use of antiarrhythmic drugs (AADs) was discontinued five elimination half-lives before the procedure. Transesophageal echocardiogram (TEE) or cardiac CT angiogram was used to confirm the absence of a thrombus in the LA 1 day before the procedure.

**Figure 1:**
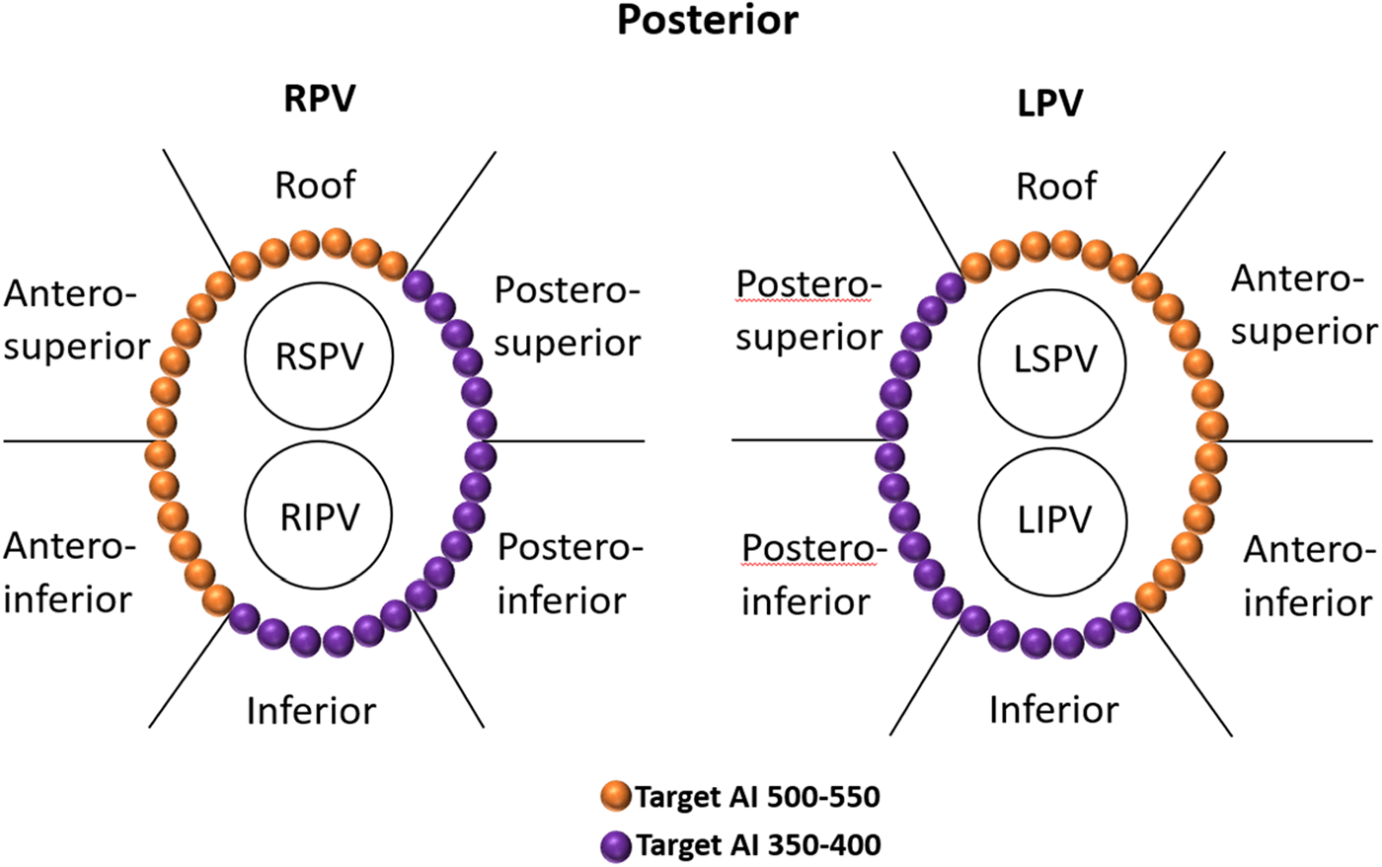
Regional definitions of right and left CCLs and schematic diagrams of target AIs in different regions of CCLs. The target AI values were 500–550 for the roof and anterior walls and 350–400 for the posterior and inferior walls. CCLs, continuous circular lesions; AI, ablation index.

**Figure 2:**
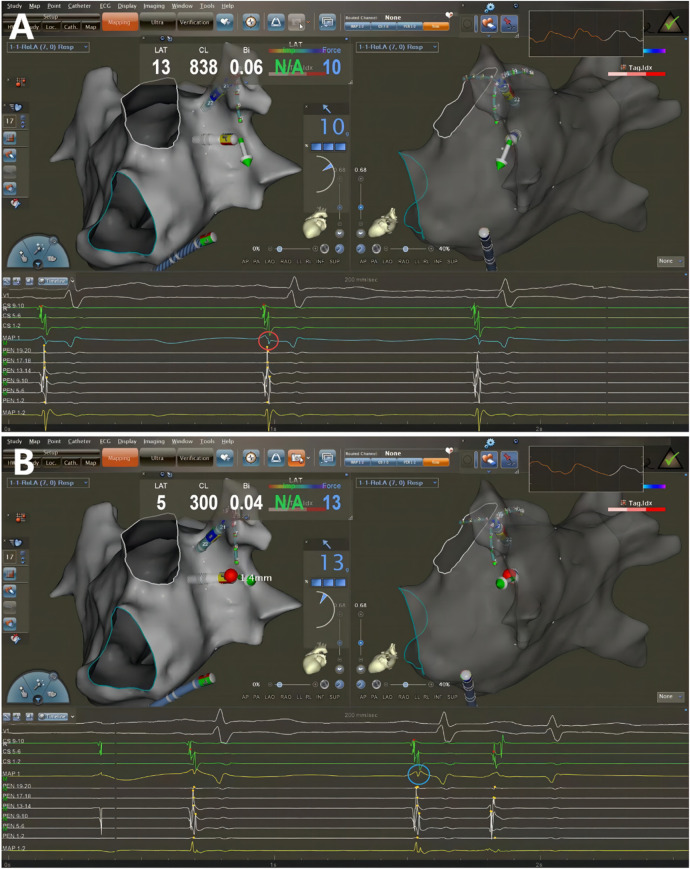
Catheter ablation guided by AI combined with UP-EGM. Every point in the CCLs was ablated following CLOSE protocol. The UP-EGM for every ablation point was also recorded for comparison. **(A)** Before ablation, the UP-EGM of the point showed a negative component, Rs pattern (in the red circle). **(B)** After ablation, UP-EGMs became completely positive, rsr pattern (in the blue circle). AI, ablation Index; UP-EGM, unipolar electrogram; CCLs, continuous circular lesions.

The patients were positioned supine and deep sedated for the ablation procedure. A single dispersive electrode pad was applied throughout the intervention and located on the lower back. A steerable catheter equipped with multiple electrodes was introduced into the coronary sinus (CS) through the route of the left femoral vein. Subsequent to the execution of a bilateral transseptal puncture utilizing access via the right femoral vein, anticoagulant therapy with heparin was commenced to aim for an activated clotting time (ACT) within the range of 250–350 s. Through transseptal access, a nonsteerable sheath (Swarts™, Abbott, MN, USA) was placed into the LA. Then, the mapping catheter (Pentaray, Biosense Webster Inc., CA, USA) and an ST 7F unidirectional irrigated tip sensor force ablation catheter (Thermocool SmartTouch®, Biosense Webster Inc., CA, USA) were advanced into the LA via the above sheaths. A geometry mapping system (Carto System, Biosense Webster Inc., CA, USA) was used to create a three-dimensional electroanatomic map and intracardiac EGM recordings. The bandwidth filter was calibrated to 0.5–180 Hz for capturing UP-EGMs. All Catheter ablation lines (CCLs) were established by point-to-point ablation. All CCLs were divided into six regions: the roof, antero-superior, antero-inferior, inferior, postero-inferior, and postero-superior regions (Figure 1) (6). The RF ablation setting parameters were standardized at 35 W, 43°C, and 25 ml/minute for all the regions above, with a target force of 5–15 g. Each patient underwent ablation guided by AI. Upon attaining the target AI threshold, RF energy delivery was halted, and the catheter was repositioned to the next site (depicted in Figure 2). To avoid the instability leading to an RF tag not reaching the AI target, a new RF application reaching the AI target was applied. The maximal interlesion distance between two neighboring lesions was ≤6 mm. In the absence of first-pass isolation (i.e., no isolation after completing the circle), touch-up ablation was delivered until PVI (9).

All procedures were performed under sinus rhythm (SR) as the precise evaluation of UP-EGM is less effective during AF. Therefore, if patients presented with AF at the beginning of or during the procedure, they would receive cardioversion. The UP-EGMs were recorded in real time with a Carto System® at a sweep speed of 200 mm/s during ablation. The AI value at which the UP-EGM completely became positive was recorded. The endpoint for every RF point was the achievement of the recommended AI value, i.e., 450–500 in the anterior wall and 350–400 in the posterior wall (Figure 1) (3).

Pentaray catheter was used to verify the PVI (entrance and exit block) with the absence of any PV or LA potential in the PV antral ablation area, and a complete bidirectional block was systematically confirmed by pacing (output of 20 mA at a pulse width of 2 ms) for each bipole of the Pentaray catheter (2, 6). Thirty minutes after PVI, the complete bidirectional blocks were rechecked for each PV. Following a thirty-minute interval after PVI, the complete bidirectional conduction blocks for each PV was reassessed. In instances of PV reconnection, additional RF was applied to reisolate the PVs among the gaps. at the end of the procedures. AF induction attempts were carried subsequent to the completion of these procedures. Supplemental RF applications was selectively applied in response to identified AF triggers. The cavotricuspid isthmus was ablated only in cases of documented common flutter.

### 2.3 Postablation management and follow-up

Anti-arrhythmic drugs (AADs) and oral anticoagulants (novel oral anticoagulants or vitamin K antagonists) were prescribed for 3 months. Subsequently, the AADs were discontinued, and anticoagulants were administered according to the CHA2DS2-VASc score. Patients underwent routine monitoring in the outpatient clinic at intervals of 3-, 6-, and 12-months postablation. Standard 12-lead ECGs were conducted, complemented by either 24-hour or 1-week Holter monitoring, as part of the standard protocol for each subsequent visit. Atrial tachycardia (AT)/AF recurrence was defined as any episode lasting >30 s (either symptomatic or asymptomatic) after a 3-month blanking period (2).

### 2.4 Statistical analysis

Continuous variables adhering to a normal distribution are presented as mean (standard deviation), with comparisons made using the *t* test. Nonnormally distributed continuous variables are detailed by median (interquartile ranges) and compared using the Mann‒Whitney *U* test. Categorical variables were compared using the chi-square test or Fisher's exact test as appropriate. The Kaplan–Meier method was used to calculate the rate of freedom from AF during the 12-month follow-up period. *P* values <0.05 were considered to indicate statistical significance. All the statistical analyses were performed using SPSS Statistics 25 (IBM Corporation, Armonk, NY, USA).

## 3 Results

### 3.1 Baseline characteristics

A total of 50 consecutive patients (aged 61.8 ± 8.5 years; 52% male) were enrolled in the study. The population characteristics are shown in Table 1. The average left ventricular ejection fraction was 65%, the average LA size was 36.2 mm, and the median CHA2DS2-VASc score was 2.0.

**Table 1:** Baseline characteristics.

Variables	
Age, years	61.8 ± 8.5
Male, *n* (%)	26 (52%)
Body mass index	23.7 ± 3.0
History of AF, months	12.0 (1.0, 36.0)
CHA2DS2-VASc score	2 (1, 3)
HAS-BLED score	1 (1, 2)
Comorbidity	
Hypertension, *n* (%)	32 (64%)
Diabetes mellitus, *n* (%)	6 (12%)
Coronary artery disease, *n* (%)	3 (6%)
Previous TIA/stroke, *n* (%)	5 (10%)
Thyroid dysfunction, *n* (%)	5 (10%)
OSAS, *n* (%)	7 (14%)
Echocardiography	
LA diameter, mm	36.2 ± 5.5
LVEF,%	65.0 ± 5.1
Anticoagulant therapy	
Warfarin	2 (4%)
NOACs	48 (96%)

LA, left atrial; LV, left ventricular ejection fraction; OSAS, obstructive sleep apnea syndrome; NOACs, novel oral anticoagulants.

### 3.2 Index AF ablation procedure

The data of the index AF ablation procedure are shown in Table 2. The time to achieve UP-EGM modification in the posterior wall and the anterior wall are shown in Table 3. The procedural duration was 112 min; the median x-ray exposure time was 5.8 min; the average ablation time was 31.4 min; and the average ablation energy delivered was 59.3 kJ. One patient (1.7%) had a pseudoaneurysm. No esophageal injury, phrenia, nerve injury, cardiac tamponade, or stroke occurred.

**Table 2:** AF ablation procedures.

Variables	
Procedural duration, min	112 (103, 116)
X-ray exposure, min	5.8 (4.6, 7.8)
Ablation time, min	31.4 ± 4.1
Ablation energy delivered, kJ	59.3 ± 6.8
Mean impedance decrease, Ω	11 (10, 12)
Mean CF, g	11 (10, 12)
Required targeting of the carina regions for PVI completion, *n* (%)	9 (18%)
The first-pass PVI, *n* (%)	48 (96%)
Acutely PVI, *n* (%)	50 (100%)
Additional ablation after PVI	
Cavotricuspid isthmus ablation, *n* (%)	3 (6%)

**Table 3:** The average time to AI_UP-EGM(+)_ and AI_END_ of six regions in the RPV and LPV.

Region	Time to AI_UP-EGM(+)_, s	Time to AI_END_, s
RPV		
Antero-superior	10.3 ± 1.6	36.9 ± 6.4
Antero-inferior	11.4 ± 2.1	34.8 ± 7.3
Roof	9.7 ± 1.8	32.2 ± 6.9
Inferior	7.2 ± 1.7	21.3 ± 6.3
Postero-superior	6.8 ± 1.6	23.3 ± 5.2
Postero-inferior	6.9 ± 1.9	26.3 ± 7.1
LPV		
Antero-superior	12.3 ± 3.6	37.4 ± 7.9
Antero-inferior	10.9 ± 2.6	41.5 ± 9.3
Roof	10.2 ± 3.8	30.7 ± 6.6
Inferior	7.6 ± 2.1	23.3 ± 4.6
Postero-superior	6.9 ± 2.1	26.6 ± 5.7
Postero-inferior	6.3 ± 1.9	25.3 ± 6.1

RPV, right pulmonary vein; LPV, left pulmonary vein.

### 3.3 First PVI rate

PVI verified with bidirectional conduction block was achieved in the entire cohort of patients. Nine patients (18%) received targeting of the PV carina despite circular lesion creation around the ipsilateral PV ostia with complete positive UP-EGMs. Thirty minutes after PVI, two patients presented PV reconnection with one gap indicating that the first PVI rate was 96%. All gaps were in the PV carina. In two patients, the PVs were reisolated by targeting the earliest PV potentials recorded by the Pentaray catheter. In two cases, the PVs underwent re-isolation by specifically directing ablation towards the initial PV electrical signals detected by the Pentaray catheter. Immediately after the PVs were reisolated, the negative component of UP-EGM in the gaps became positive.

### 3.4 AI values and UP-EGM elimination

**Figure 3:**
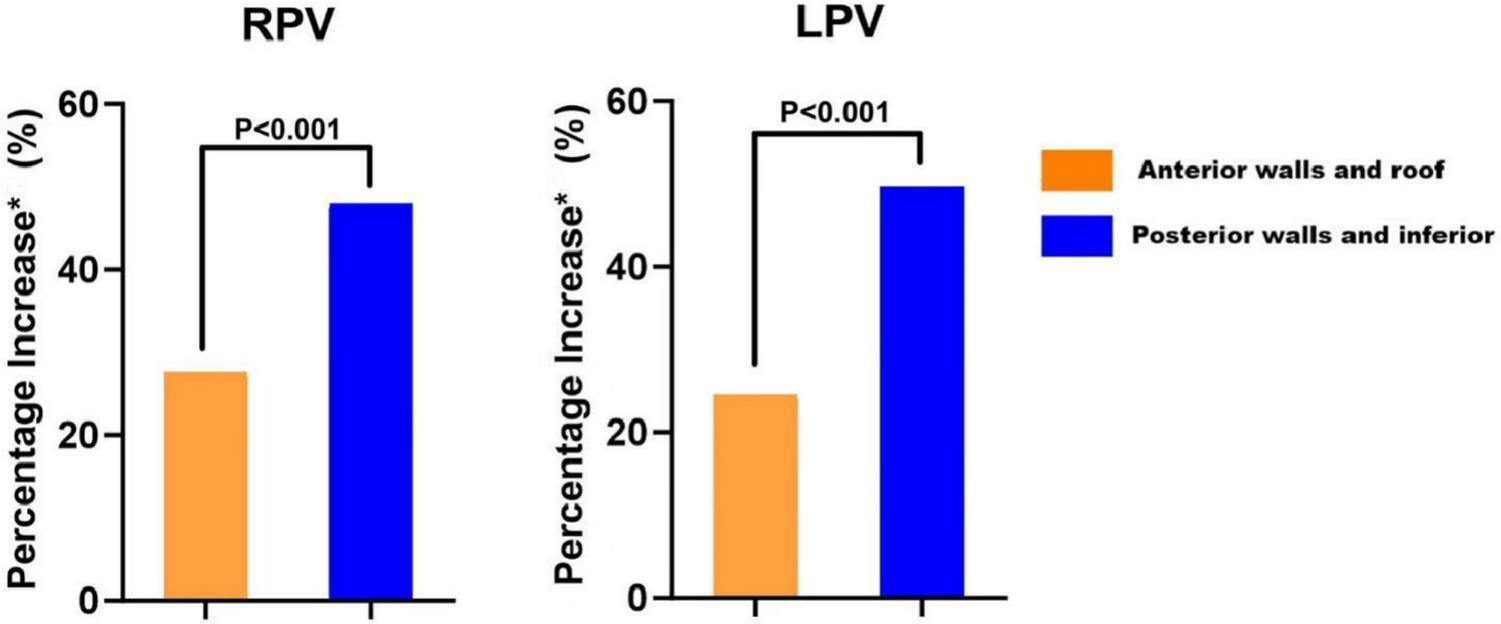
Comparison of percentage increase between anterior and posterior walls in RPV and LPV. Percentage increase* indicates the rate of increase from AI_END_ to AI_UP-EGM_. RPV, right pulmonary vein; LPV, left pulmonary vein.

The number of lesion points in each region ranged from 4 to 9. A total of 2 954 lesion points were analyzed. The demographic data of AI_UP-EGM(+)_ and AI_END_ in each region of PVs are shown in Table 4. The average AI_UP-EGM(+)_ in the posterior wall and the anterior wall was 420.9 and 267.4, respectively. The average AI_END_ in the posterior wall and the anterior wall was 524.3 and 393.9. The percentage increase in the posterior wall of right PV and left PV was 47% ± 12% and 49% ± 12%, while that in the anterior wall of right PV and left PV was 28% ± 9% and 22% ± 13% (shown in Table 5 and Figure 3), thus indicating significant differences between the posterior and anterior walls.

**Table 4:** The average AI_UP-EGM(+)_ and AI_END_ of six regions in the RPV and LPV.

Region	AI_UP-EGM(+)_	AI_END_	*D*-value*	Percentage increase** (%)
RPV				
Antero-superior	408.65 ± 24.58	533.75 ± 14.62	125.10 ± 23.23	30.9 ± 7.3
Antero-inferior	410.96 ± 29.37	531.10 ± 18.42	120.15 ± 26.57	29.8 ± 9.5
Roof	426.44 ± 23.91	520.02 ± 14.63	93.58 ± 14.63	22.2 ± 7.1
Inferior	258.85 ± 20.89	394.26 ± 11.05	135.41 ± 23.11	53.2 ± 11.2
Postero-superior	270.79 ± 15.92	397.90 ± 13.93	127.11 ± 18.40	47.4 ± 9.0
Postero-inferior	281.78 ± 23.31	391.08 ± 9.28	109.30 ± 24.10	39.7 ± 11.9
LPV				
Antero-superior	427.07 ± 24.62	526.09 ± 16.57	99.02 ± 27.53	23.6 ± 8.5
Antero-inferior	429.10 ± 13.91	518.43 ± 11.97	89.33 ± 18.02	20.9 ± 4.9
Roof	421.25 ± 19.21	516.17 ± 17.25	92.96 ± 16.78	22.0 ± 4.5
Inferior	261.31 ± 23.78	393.54 ± 13.60	132.23 ± 24.39	51.8 ± 13.2
Postero-superior	270.75 ± 22.67	392.63 ± 14.08	116.24 ± 20.50	42.7 ± 9.9
Postero-inferior	255.54 ± 21.88	399.00 ± 16.68	138.28 ± 17.97	54.8 ± 10.4

*D*-value *indicates the difference between AI_UP-EGM(+)_ and AI_END_. Percentage increase **indicates the percentage of *D*-values relative to the AI_UP-EGM(+)_ group. RPV, right pulmonary vein; LPV, left pulmonary vein.

**Table 5:** The average *D*-value and percentage increase in RPV and LPV.

	Posterior wall	Anterior wall	*P* value
RPV			
AI_UP-EGM(+)_	270.47 ± 22.23	415.35 ± 27.08	0.331
AI_END_	394.41 ± 27.01	528.29 ± 16.97	<0.001
*D*-value*	123.94 ± 24.44	112.94 ± 28.50	0.062
Percentage increase**	47% ± 12%	28% ± 9%	<0.001
LPV			
AI_UP-EGM(+)_	264.41 ± 23.76	426.46 ± 23.76	0.369
AI_END_	393.32 ± 15.03	520.23 ± 15.91	0.003
*D*-value*	128.91 ± 22.96	93.77 ± 21.56	0.042
Percentage Increase**	49% ± 12%	22% ± 6%	<0.001

*D*-value *indicates the difference between AI_UP-EGM(+)_ and AI_END_. Percentage increase **indicates the percentage of *D*-values relative to the AI_UP-EGM(+)_ group. RPV, Right pulmonary vein; LPV, Left pulmonary vein.

### 3.5 Follow-up

**Figure 4:**
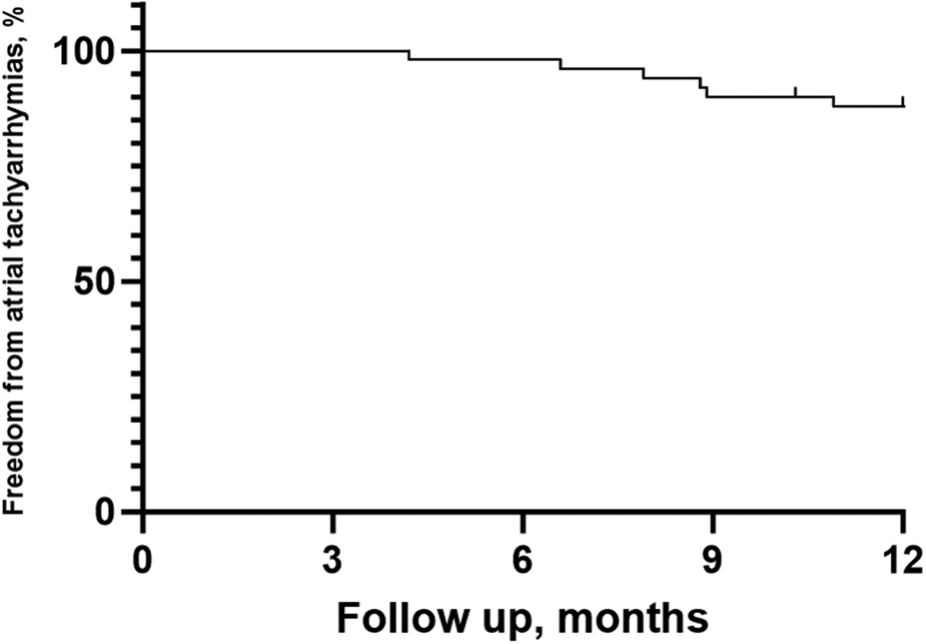
Kaplan–Meier survival curve for atrial arrhythmia recurrence in the study.

After a mean follow-up period of 11.30 ± 2.10 months, 44 patients (88%) remained in SR without AADs (Figure 4). Among the 6 patients who experienced AF recurrence, four patients experienced PAF recurrence, while 2 experienced AT recurrence. One patient underwent a second PVI procedure demonstrating PV reconnection in the right roof of the RPV and atrial tachycardia originating from the mitral isthmus. There was no PV reconnection in the left PV carina, posterior walls or roof.

## 4 Discussion

### 4.1 Main findings

The main findings of the present investigation were as follows. (1) The AI values when UP-EGM became completely positive were lower than recommended AI values in all regions of the PVs. (2) The average percentage increase from AI_UPEGM(+)_ to AI_END_ in the posterior walls and inferior walls was greater than that in the anterior walls and roof.

### 4.2 UP-EGM-guided AF ablation

Previous studies have shown that CLOSE-guided PVI improves procedural and 1-year outcomes while shortening procedure time; subsequently, RF delivery is stopped at an AI of 550 in the anterior wall and at an AI of 450 in the posterior wall (2). Other studies have also shown that AI-guided high-power RFCA is safe and effective in treating PAF patients while reducing the procedure time (9). These studies considered the difference in the thickness of the atrium but ignored the difference between individuals. UP-EGM can reflect TLs at any time during ablation. Our previous study also showed that PVI was guided by UP-EGM with the above ablation procedure; subsequently, the success rate was 86% after a mean follow-up of 19 ± 5 months (6). For high-power ablation strategies, high-power (40–50 W) PVI guided by UP-EGM was also proven to be safe and effective, with 90% sinus rhythm maintenance at 12 months (10). Ejima et al. confirmed that UP-EGM modification using high power (50 W) has a shorter procedure time and better outcomes (11). However, most of the centers use a conventional, low-power long-duration approach for PVI procedures. In this study, our 12-month follow-up results are consistent with those of a previous study, and no complications occurred after the operation procedure, which also confirmed that AI-guided UP-EGM ablation is safe and effective for PAF patients.

### 4.3 The advantages of UP-EGM

Otomo et al. demonstrated that UP-EGM modification during PVI using RF energy by eliminating the negative deflection of UP-EGM was associated with TLs formation in a porcine model, and TLs with a nonparallel or parallel catheter orientation consistently exhibited a complete abolition of the negative deflection with a mild attenuation of the positive deflection, whereas those recorded from non-TLs did not (4). Therefore, the elimination of the negative component on UP-EGM should not be influenced by the orientation of the catheter. In addition, the ablation duration for eliminating the negative component of UP-EGM reflects the thickness of the ablation point (5). After TLs were confirmed by mapping, UP-EGM became completely positive. Our previous study explored the different types of UP-EGM after PVI and revealed that PVI guided by UP-EGM with a complete positive pattern in PAF patients is reliable (6). These are the advantages of UP-EGM. In this study, the patterns of UP-EGM at the end of ablation were consistent with those of previous studies, and the 12-month follow-up data were also similar to those of previous studies, which also reflects the advantages of UP-EGM.

### 4.4 The percentage increase from AI_UP-EGM(+)_ to AI_END_

Previous studies have confirmed the advantages and individuation of PVI with the guidance of UP-EGM. A 5-second extension of ablation after the UP-EGM became completely positive was proven to be necessary and effective for creating TLs in previous lesions (8). However, similar to the CLOSE protocol, the 5-second extension of ablation is not a proper quantitative evaluation because the thickness and atrial tissue differ in different regions and individuals. In this study, the average percentage increase from AI_UP-EGM(+)_ to AI_END_ was 22% and 28% in the anterior walls and roof, respectively, but the average percentage increase in the posterior walls and inferior walls was 47% and 49%, respectively. Our study used the percentage increase in the AI instead of the exact value of the AI to offset the difference between individuals. After 30 min of observation, the UP-EGM in the anterior wall was still positive, suggesting that the TLs with a percentage increase of 22%–28% were reliable in the posterior wall and roof. The results of our 12-month follow-up are similar to those of previous studies, and there was no reconnection of the anterior or roof walls in patients who underwent a second PVI procedure, thus indicating that the percentage increase in the AI following the anterior walls and roof is stable. Overall, these findings show that at markedly lower AIs than the generally recommended AI values, lesions transmurally guided by the UP-EGM were achieved in the posterior and inferior walls. The RF application have improved its safety with the development of catheter and mapping system, but excessive ablation in the posterior and inferior walls may still occur in clinical practice. Excessive ablation increases the risk of serious complications such as atrioesophageal fistula, pericardial tamponade, cardiogenic stroke and pulmonary vein stenosis (12, 13). Although current strategies for PVI have been proven to be safe, achieving TLs with less radiofrequency energy is safer. The results in this study showed that the ablation in posterior wall might be excessive. We think that ablation guided by UP-EGM may have safety implications by limiting the amount of RF energy delivered.

There have been no similar studies on the ratio or percentage increase between the AI at the time of UP-EGM modification and the AI at the end of ablation. A 22%–28% percentage increase in the AI is sufficient in the posterior walls. In future operation procedures, the target AI values in the posterior walls and inferior walls can be reduced according to this study, but further research is necessary to validate this finding.

## 5 Limitations

The current study had several limitations. First, this was a single-center investigation with a limited sample size. Second, only one patient with atrial tachycardia/AF recurrence underwent a second PVI procedure. The mechanism of recurrence is unclear. Third, the follow-up period was only 1 year. The results of long-term follow-up should be further explored. Forth, The RF ablation setting was 35w, and results of this study may not be applied to high-power ablation strategy. Fifth, this is a pilot study. The results and its clinical implication should be proven in further study.

## 6 Conclusion

Ablation index-guided catheter ablation combined with unipolar electrograms is safe and effective in patients with paroxysmal atrial fibrillation. The current recommended AI values might be excessive in posterior walls and inferior, but this remains to be proven in future research.

## Data Availability

The datasets presented in this article are not readily available due to patient privacy. Requests to access the datasets should be directed to Yijun Sun, sunyj1999@126.com.
